# Hierarchical Dynamic Causal Modeling of Resting-State fMRI Reveals Longitudinal Changes in Effective Connectivity in the Motor System after Thalamotomy for Essential Tremor

**DOI:** 10.3389/fneur.2017.00346

**Published:** 2017-07-20

**Authors:** Hae-Jeong Park, Chongwon Pae, Karl Friston, Changwon Jang, Adeel Razi, Peter Zeidman, Won Seok Chang, Jin Woo Chang

**Affiliations:** ^1^BK21 PLUS Project for Medical Science, Yonsei University College of Medicine, Seoul, South Korea; ^2^Department of Nuclear Medicine, Severance Hospital, Yonsei University College of Medicine, Seoul, South Korea; ^3^Center for Systems Brain Sciences, Institute of Human Complexity and Systems Science, Yonsei University, Seoul, South Korea; ^4^Department of Cognitive Science, Yonsei University, Seoul, South Korea; ^5^The Wellcome Trust Centre for Neuroimaging, University College London, London, United Kingdom; ^6^Monash Institute of Cognitive and Clinical Neurosciences, Monash Biomedical Imaging, Monash University, Clayton, VIC, Australia; ^7^Department of Electronic Engineering, NED University of Engineering and Technology, Karachi, Pakistan; ^8^Department of Neurosurgery, Yonsei University College of Medicine, Seoul, South Korea

**Keywords:** thalamotomy, essential tremor, brain network, effective connectivity, dynamic causal modeling

## Abstract

Thalamotomy at the ventralis intermedius nucleus for essential tremor is known to cause changes in motor circuitry, but how a focal lesion leads to progressive changes in connectivity is not clear. To understand the mechanisms by which thalamotomy exerts enduring effects on motor circuitry, a quantitative analysis of directed or effective connectivity among motor-related areas is required. We characterized changes in effective connectivity of the motor system following thalamotomy using (spectral) dynamic causal modeling (spDCM) for resting-state fMRI. To differentiate long-lasting treatment effects from transient effects, and to identify symptom-related changes in effective connectivity, we subject longitudinal resting-state fMRI data to spDCM, acquired 1 day prior to, and 1 day, 7 days, and 3 months after thalamotomy using a non-cranium-opening MRI-guided focused ultrasound ablation technique. For the group-level (between subject) analysis of longitudinal (between-session) effects, we introduce a multilevel parametric empirical Bayes (PEB) analysis for spDCM. We found remarkably selective and consistent changes in effective connectivity from the ventrolateral nuclei and the supplementary motor area to the contralateral dentate nucleus after thalamotomy, which may be mediated *via* a polysynaptic thalamic–cortical–cerebellar motor loop. Crucially, changes in effective connectivity predicted changes in clinical motor-symptom scores after thalamotomy. This study speaks to the efficacy of thalamotomy in regulating the dentate nucleus in the context of treating essential tremor. Furthermore, it illustrates the utility of PEB for group-level analysis of dynamic causal modeling in quantifying longitudinal changes in effective connectivity; i.e., measuring long-term plasticity in human subjects non-invasively.

## Introduction

Thalamotomy at the ventralis intermedius nucleus (Vim) is an effective treatment for essential tremor (1–3). A focal lesion of the Vim by thalamotomy is known to induce progressive changes at the circuit level of the motor system. These progressive changes have been detected, not only in structural connectivity at remote regions—measured with diffusion tensor imaging (DTI) (4)—but also in the inter-regional temporal synchrony (i.e., functional connectivity) of resting-state functional magnetic resonance imaging (rs-fMRI) signals (5). Jang and colleagues (5) have shown thalamotomy-induced longitudinal changes of resting-state functional connectivity in both the whole brain network and at the level of the motor circuit. They suggested that alterations of resting-state functional connectivity over time involve a mixture of symptom-related (sustained) treatment effects and non-symptom-related (or indirectly related) transient effects.

Resting-state functional connectivity, however, is usually defined by a Pearson correlation between regional fluctuations in hemodynamic activity and does not characterize directed coupling among brain regions [for review, see Ref. (6)]. Therefore, if we are to understand the mechanisms by which thalamotomy exerts its effects on the motor circuitry in essential tremor; quantitative estimates of directed effective connectivity are required. Effective connectivity refers to the causal influence that one neural system exerts over another, either at a synaptic or population level (7).

In the present study, we attempted to identify long-lasting symptom-related changes in the resting-state (intrinsic) effective connectivity of the motor circuitry following a thalamotomy. To estimate intrinsic effective connectivity, we used spectral dynamic causal modeling (DCM) (8), which estimates effective connectivity among brain regions in terms of neuronal coupling and subsequent hemodynamic responses, using cross-spectral summaries of rs-fMRI data. This method has been evaluated in a construct validation study (9, 10), aging study (11), and an optogenetic fMRI study (12).

To differentiate long-lasting treatment effects from transient effects on effective connectivity and to identify symptom-related changes in effective connectivity, we applied spectral DCM (spDCM) to longitudinal rs-fMRI data acquired 1 day prior to the thalamotomy and 1 day, 7 days, and 3 months after the thalamotomy. In conventional procedures, investigations of the transient changes that occur following a thalamotomy are hindered by the inherent limitations of invasive techniques, such as inevitable damage to non-target regions and potential side effects due to opening the cranium. To overcome this problem, we used data from clinical cases of thalamotomies that were performed with a recently established MRI-guided high-intensity focused ultrasound (MRgFUS) thermal ablation technique to treat essential tremors (13–17).

Using spDCM to model effective connectivity in each session of each patient calls for a method that can estimate any longitudinal changes at the group level. Thus, we propose a method for group-level longitudinal analysis of effective connectivity. For this purpose, we adopted parametric empirical Bayes (PEB) for DCM (18, 19), which models random (between-session and -subject) effects on coupling parameters that are estimated at the first (within-session and -subject) level. In contrast to random effects on models *per se* (20), the PEB scheme allows for random effects on connection strengths between sessions or subjects; i.e., random effects on model parameters as opposed to models. We extended the PEB approach by conducting a two-level PEB analysis of the between-session and between-subject group effects. We also attempted to identify the effective connectivity that was associated with the longitudinal symptom changes. In doing so, we will illustrate the utility of PEB for DCM in quantifying group-level changes in longitudinal effective connectivity; i.e., long-term plasticity that is conserved over subjects.

## Materials and Methods

### Subjects and MRgFUS Thalamotomy

**Figure 1:**
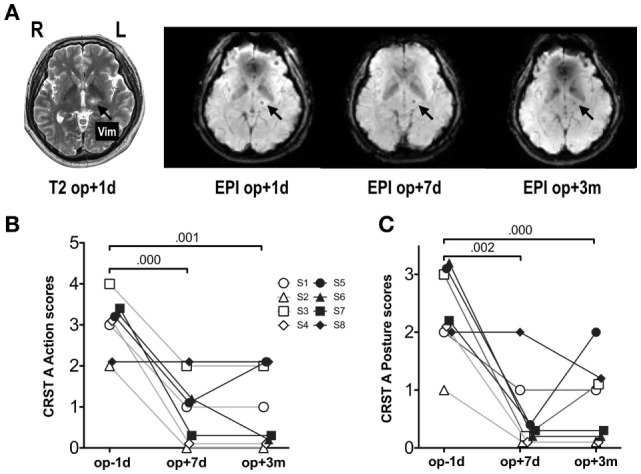
MRI and fMRI scans and symptom severity measures before and after thalamotomy. **(A)** Lesion locations in T_2_-weighted images and fMRI echo-planar imaging (EPI) after MRgFUS thalamotomy in a patient. The target lesion is the left ventralis intermedius nucleus (Vim) of the thalamus (arrow). **(B)** Clinical Rating Scale for Tremor Part A (CRST A) action scores. **(C)** CRST A posture scores. Repeated-measures ANOVA for both scores showed significant session effects (*p* < 0.001). The numbers over the lines indicate *p*-values following *post hoc* analysis. op − 1d, op + 1d, op + 7d, and op + 3m indicate 1 day before treatment, 1 day, 7 days, and 3 months after treatment. S1–S8 refers to subjects 1–8.

The data used in this study have been described previously (5). Briefly, eight patients with essential tremor (mean age 65 years; one female) underwent left thalamotomy by thermally ablating a target in the ventralis intermedius nucleus (Vim) of the thalamus using MRgFUS. All MRgFUS procedures were performed using ExAblate 4000 (InSightec, Tirat Carmel, Israel), which is combined with a 3 T MRI (GE, Milwaukee, WI, USA). MRgFUS destroys tissues by focusing a high-energy beam on the Vim of the thalamus and raising its temperature up to the range of 55–60°C. This ablation procedure was conducted without cranium-opening under the guidance of real-time MRI temperature mapping for targeting. Details of the procedure can be found elsewhere (13). All patients completed 4 sessions of resting-state fMRI scanning: before treatment (op − 1d), 1 day after treatment (op + 1d), 7 days after treatment (op + 7d), and 3 months after treatment (op + 3m). The degree of tremor was evaluated using the Clinical Rating Scale for Tremor Part A (CRST A) (21). The right hand CRST A action score represents the degree of tremor for patients moving their hands, while CRST A posture score represents the tremor score without moving. All patients received a standard clinical and imaging workup as part of the study’s baseline requirements. In the current study, we continued the same medication, if a patient was on any prescribed medication before treatment. All patients provided written informed consent before procedures, and this study received full ethics approval from the Korean Food and Drug Administration and Institutional Review Board of Yonsei University Severance Hospital and the Declaration of Helsinki (World Medical Association, 1964, 2008). Figure 1 shows an example of the target ablation region and its longitudinal changes in fMRI data.

### Data Acquisition and Processing

Resting-state fMRI data were acquired axially with $T_{2}^{*}$-weighted single shot echo-planar imaging (EPI) sequences in a 3.0-T GE 750 MRI scanner (GE, Milwaukee, WI, USA) using a 16 channel head neck spine array coil with the following parameters: voxel size, 1.88 mm × 1.88 mm × 4.5 mm; slice number, 30 (interleaved); matrix, 128 × 128; slice thickness, 4 mm; repetition time, 2,000 ms; echo time, 30 ms; flip angle = 90°; and field of view, 240 mm × 240 mm. Each 330-s scan produced 165 fMRI images. Foam pads were used to reduce head motion during EPI data acquisition. Subjects were instructed to keep their eyes closed, while avoiding sleeping or specific thinking.

Spatial preprocessing of fMRI data was conducted using statistical parametric mapping (SPM12, http://www.fil.ion.ucl.ac.uk/spm/, Wellcome Trust Centre for Neuroimaging, London, UK) (22). All EPI data underwent standard preprocessing steps including correction of acquisition time delays between different slices, correction for head motion by realigning all consecutive volumes to the first image of the session, and non-linear coregistration of T1-weighted image to the first EPI data. Coregistered T1-images were used to spatially normalize functional EPI into the Montreal Neurological Institute (MNI) template space using non-linear transformation in SPM12. All registration steps were confirmed visually and semiautomatically adjusted in the case of a misregistration.

**Figure 2:**
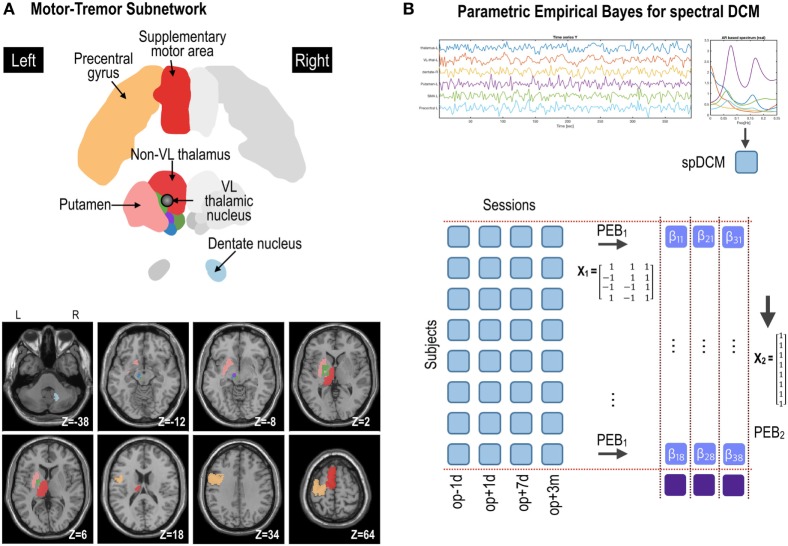
Procedures for parametric empirical Bays (PEB) effective connectivity analysis of the motor subnetwork **(A)**. Spectral dynamic causal modeling (spDCM) was applied to each patient’s resting-state fMRI data at four time points: op − 1d, op + 1d, op + 7d, and op + 3m (1 day before operation, 1 day after operation, 7 days after operation, and 3 months after operation, respectively) **(B)**. The motor subnetwork includes the precentral gyrus, supplementary motor areas, putamen, ventrolateral (VL) thalamic nucleus, and non-VL thalamic nuclei in the left hemisphere, and right dentate nucleus in the cerebellum. The coordinate in panel **(A)** (e.g., *Z* = −38) is the Montreal Neurological Institute template space. L and R indicate left and right. For the group-level analysis, PEB analysis was applied at two levels; (1) PEB_1_ across four sessions with a regressor of a transient effect ([1 −1 −1 1]), a regressor of a sustained treatment effect ([1 1 −1 −1]), and a baseline ([1 1 1 1]); (2) PEB_2_ across (eight) patients with a regressor of [1 1 1 1 1 1 1 1]^T^ to model group effects across subjects.

We extracted fMRI time series for each patient in each session from six brain regions in the standard anatomical (atlas) space to examine the motor-tremor network. The six brain regions were the left precentral gyrus, left supplementary motor area (SMA), left ventrolateral (VL) nuclei of the thalamus (VL, the ablation target region), left thalamus (except for the VL nuclei), left putamen, and right dentate nucleus (Figure 2A). The precentral gyrus, thalamus, and dentate nucleus are known to be involved in the tremor circuit (23). The thalamus, putamen, and cortical motor areas were extracted from the Automated Anatomical Labeling map (24). The ablation target VL region was extracted manually by identifying the location of the ablation in T_2_-weighted images. We manually delineated the dentate nucleus of each patient, according to the method detailed by Tellmann and colleagues (25). Regional time series were summarized as the principal eigenvariate of voxel time series within each region of interest.

After discarding the first five scans to ensure magnetic equilibration, we preprocessed fMRI time series by regressing out the effects of six rigid motions and their derivatives and the three principal components of signal fluctuations in white matter and cerebrospinal fluid masks. fMRI time series were detrended by linear and quadratic repressors and low pass filtered up to 0.009 Hz. We did not remove higher frequencies (>0.1 Hz), as they are known to contain meaningful information in resting-state studies (26, 27).

### Effective Connectivity Estimation Using Spectral DCM

To estimate connectivity among the six brain regions, we applied spectral DCM (spDCM) (8) (implemented in SPM12) to resting-state fMRI. For a directed asymmetric network model with six nodes (or regions) and observed blood oxygenation level dependent (BOLD) signals, spDCM estimates the parameters of the following two generative models; a stochastic model of neuronal dynamics and a hemodynamic response model *h*.

(1) $$\begin{array}{l} \dot{x}=Ax+v\\ y=h(x,{\text{θ}}_{h})+e,\;e∼N(0,\text{Σ}) \end{array}$$

where *x* represents the hidden neural state for each region and the matrix *A* represents the intrinsic effective connectivity among nodes. Endogenous or intrinsic (neural) fluctuations are denoted by *v*. The measured BOLD signal *y* is modeled as a non-linear hemodynamic response function *h* of neuronal states *x* and parameters θ*_h_* [based on the Balloon model (28)] with an additive observation noise *e*. spDCM simplifies the two generative models by replacing the original timeseries with their second-order statistics (i.e., cross spectra). This means, instead of estimating time varying hidden states, spDCM estimates their covariance, which is time invariant. Then, we simply need to estimate the covariance of the random fluctuations; where a scale free (power law) form for the state noise is used—motivated from previous works on modeling neuronal fluctuations (29–31). For more detailed description of spectral DCM, please refer to Ref. (8).

### PEB Estimation of Transient and Treatment-Related Effective Connectivity

We conducted group-level inference for spDCM using an empirical Bayesian approach with SPM12 (18, 19). This involved specifying a hierarchical model with three levels: (1) session level, (2) subject level, and (3) group level. Specifically, as illustrated in Figure 2, at the first level, we inverted spDCMs for each of the four sessions for each subject. The intrinsic connectivity of spDCMs was fully connected (see matrix *A* in Eq. 1), with one parameter for each connection. In this paper, we refer to the directed connectivity during the resting state as intrinsic connectivity, which mediates intrinsic brain networks. These connectivity parameters were modeled at the second (within subject) level in a linear model, for each subject separately, with regressors modeling transient and sustained effects across sessions. Finally, the second-level parameters from each subject were modeled at the third level in a linear model with regressors representing commonalities and differences across subjects (19). Within this hierarchy, the first-level models were spDCMs, whereas the second- and third-level models were (Bayesian) general linear models. This hierarchical parametric empirical base model can be described with the following series of equations:

(2) $$\begin{array}{l} y_{ij}=\text{Γ}({\text{θ}}_{ij}^{\left(1\right)})+\varepsilon_{ij}^{\left(1\right)},\;\varepsilon_{ij}^{\left(1\right)}∼N(0,{\text{Σ}}^{\left(1\right)})\\ {\text{θ}}_{ij}^{\left(1\right)}=X_{1}{\text{β}}_{j}^{\left(2\right)}+\varepsilon_{j}^{\left(2\right)},\;\varepsilon_{j}^{\left(2\right)}∼N(0,{\text{Σ}}^{\left(2\right)})\\ {\text{β}}_{j}^{\left(2\right)}=X_{2}{\text{β}}^{\left(3\right)}+\varepsilon^{\left(3\right)},\;\varepsilon^{\left(3\right)}∼N(0,{\text{Σ}}^{\left(3\right)}) \end{array}$$

The first line of Eq. 2 states that the time series data from the *i*th session and *j*th subject *y_ij_* were generated by a function *Γ*, which corresponds to Eq. 1, plus independent and identically distributed (i.i.d.) observation noise $\varepsilon_{ij}^{\left(1\right)}$. The parameters of this model ${\text{θ}}_{ij}^{\left(1\right)}$ are sampled from the *j*th second-level model, which generated the subject’s four sessions with design matrix *X*_1_ and parameters ${\text{β}}_{j}^{\left(2\right)}$, plus i.i.d. random effects $\varepsilon_{j}^{\left(2\right)}$. The final line of Eq. 2 states that the parameters for each subject β*_j_* were generated from a group-level model with design matrix *X*_2_ and parameters β^(3)^ plus a random effect ε^(3)^. Put simply, the parameters β^(3)^ represent commonalities and differences across subjects, whereas parameters β^(2)^ represent commonalities and differences across time (i.e., Sessions).

We implemented this hierarchical model in SPM12 using two levels of PEB (Figure 2): (1) PEB of DCMs across sessions for each subject with a design matrix *X*_1_ and (2) PEB of individual effect sizes (i.e., connectivity changes across sessions for each individual) across subjects, with a design matrix *X*_2_ for group inference (i.e., PEB of PEB). After estimating connectivity changes in each individual, the group averages of these treatment effects were estimated by assigning a design matrix *X*_2_ of the second stage with [1 1 1 1 … 1]^T^ (8 subjects × 1).

### PEB Estimation of Symptom-Related Effective Connectivity

We also performed PEB analysis to identify effective connectivity changes with motor-symptom scales; i.e., CRST A action and posture scores. For this purpose, we conducted the same PEB analysis as above but now replacing the longitudinal regressors with CRST A action and posture scores, measured 1 day before treatment, 7 days and 3 months after treatment.

## Results

A repeated-measures analysis of variance of the action and posture scores on the CRST A (Figures 1B,C) showed significant improvements after MRgFUS treatment [action: *F*(1.63, 11.40) = 28.00, *p* < 0.0001; posture: *F*(2, 14) = 19.08, *p* < 0.0001].

**Figure 3:**
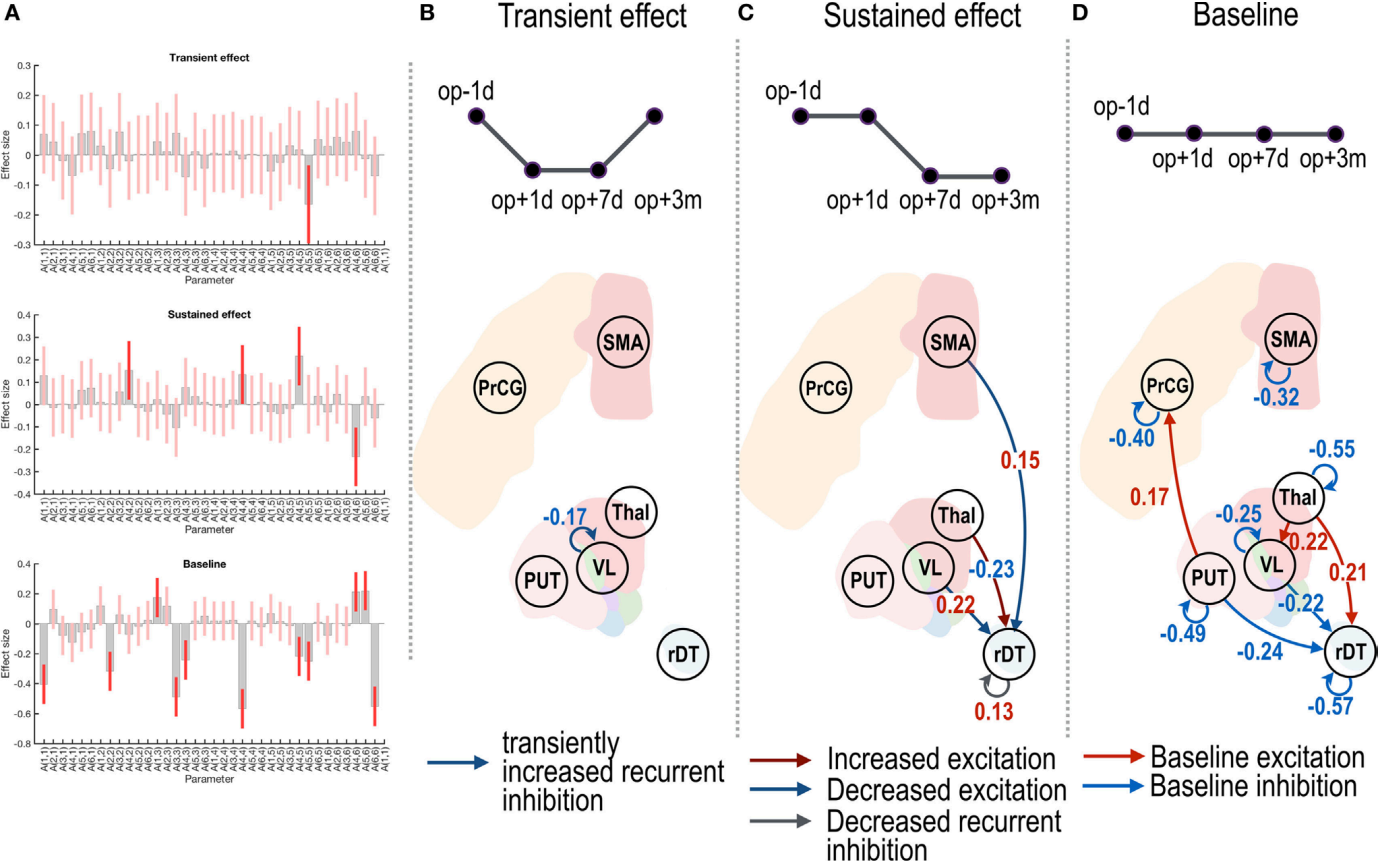
Effective connectivity changes in the motor subnetwork after thalamotomy. The intrinsic effective connectivity associated with treatment, transient, and baseline effect is displayed in the three rows of panel **(A)**, and from panel **(B)** to panel **(D)**. The session regressors for a transient effect ([1 −1 −1 1]), a treatment effect ([1 1 −1 −1]), and a baseline effect ([1 1 1 1]) are shown in the upper row in panels **(B–D)**. Effect sizes that survived a non-zero criterion with a posterior confidence of 95% are displayed in the graphs. The self-connectivity in the VL had a negative effect size **(B)**, indicating a transient change in the effective connectivity. The dark blue and dark red arrows in panel **(C)** show the positive and negative effect sizes (i.e., blue and red colored numbers) and indicate sustained changes in the effective connectivity (long-lasting increase and decrease in the effective connectivity, respectively). In contrast to the regression analysis in panels **(B,C)**, the red and blue arrows in panel **(D)** indicate the group average of baseline positive and negative effective connectivity. VL, ventrolateral thalamic nucleus; PrcG, precentral gyrus; SMA, supplementary motor area; Thal, non-VL thalamic nuclei; Put, putamen in the left hemisphere; rDT, right dentate nucleus in the cerebellum. op − 1d, op + 1d, op + 7d, and op + 3m indicate 1 day before treatment, 1 day, 7 days, and 3 months after treatment.

Figure 3 shows group-level effective connectivity changes in the motor-tremor network over sessions. We present connectivity changes that survived a non-zero criterion with a posterior confidence of 95%; i.e., an effect size of 0 was outside the confidence interval. Since only one multivariate test was used based on Bayesian statistics, there is no need to correct for multiple comparisons.

Figure 3B displays a negative effect size in the self-(or recurrent) connectivity in the VL thalamic nucleus. Note that self-connections (leading diagonal elements) in the effective connectivity matrix *A* (i.e., *A_ii_* in Eq. 1) correspond to (log) scale parameters [expressed as −1/2 exp(*A_ii_*)] during the DCM estimation such that the positive self-connectivity values indicate increased self-inhibition whereas negative self-connectivity values indicate decreased self-inhibition relative to the prior. Thus, the negative effect size with the transient regressor in Figure 3B indicates a transient increase in self-inhibition, which eventually returned to the baseline level.

In Figure 3C, the positive effect sizes (or sustained decrease) in the connectivity from the VL nucleus and SMA to the right dentate nucleus and the negative effect size (or long-lasting increase) in the connectivity from the non-VL thalamic nuclei to the right dentate nucleus suggest long-lasting changes in effective connectivity after thalamotomy. The positive effect size in the self-connectivity in the right dentate nucleus indicates a long-lasting decrease in self-inhibition (i.e., disinhibition) after treatment.

**Figure 4:**
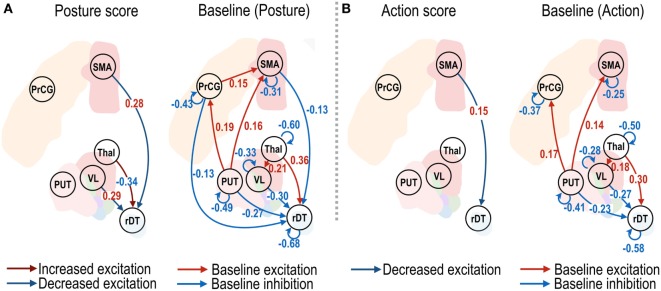
Effective connectivity of motor subnetwork associated with posture and action scores. The dark blue and dark red arrows in left panels of **(A,B)** show positive and negative effect sizes (i.e., red and blue colored numbers), indicating decreased and increased effective connectivity with reduced symptom severity. Connectivity changes that survived a non-zero criterion with a posterior confidence of 95% are displayed in the graphs. VL, ventrolateral thalamic nucleus; PrcG, precentral gyrus; SMA, supplementary motor area; Thal, non-VL thalamic nuclei; Put, putamen in the left hemisphere; rDT, right dentate nucleus in the cerebellum. op − 1d, op + 7d, and op + 3m indicate 1 day before treatment, 7 days, and 3 months after treatment.

The effective connectivity changes that were associated with changes in the clinical posture and action scores are shown in Figure 4. The changes in the effective connectivity from the VL nucleus to the right dentate nucleus, and from the SMA to the right dentate nucleus, showed positive correlations with the posture scores (Figure 4A), which suggests decreased effective connectivity predicts decreased posture scores (i.e., reduced symptom severity); in contrast, the changes in the effective connectivity from the non-VL thalamus to the right dentate nucleus showed a negative effect size; i.e., increased effective connectivity predicts decreased symptom severity. A significant positive effect size for the action scores was found only from the SMA to the right dentate nucleus (Figure 4B).

## Discussion

Enduring changes in synaptic connectivity are considered the primary therapeutic effect of thalamotomy performed to treat essential tremors. Despite the importance of connectivity analyses, studies that have investigated effective connectivity changes, particularly over time, are rarely conducted. In this paper, we applied spDCM for rs-fMRI to characterize the longitudinal changes in intrinsic effective connectivity after focal lesions of the thalamus in patients with essential tremors. We have introduced a simple and efficient way to analyze longitudinal spDCM at the group level using a multilevel PEB. The results of the current study confirmed the crucial role of the dentate nucleus in tremor symptoms and how it serves as the ultimate target of thalamotomy used to control tremor.

Essential tremors are largely attributable to neurodegeneration in the dentate nucleus (32, 33). The dentate nucleus is usually activated when the patients exhibited tremor symptoms (34). Postmortem examinations of patients with essential tremor have shown decreased fibers and neurons (35) and reduced numbers of gamma-aminobutyric acid (GABA) receptors in the dentate nucleus (36). In a previous study, Paris-Robidas and colleagues (36) argued that the reduced GABA receptors in the dentate nucleus may lead to disinhibition of cerebellar pacemaker output activity that is conveyed to the thalamus and motor cortex. This reduced inhibition in the dentate nucleus might increase local activity and alter the connectivity along the cerebello–thalamocortical circuit (37). Observations of increased functional connectivity between the Vim nucleus and the dentate nucleus in patients with tremor-dominant Parkinson’s disease also support this hypothesis (38).

Thalamotomy in the Vim nucleus might alleviate tremor symptoms by regulating connectivity as a consequence of the ablation of local Vim connections. The changes are first reflected in an increase in self-inhibition in the VL. Although this increase in self-inhibition in the VL is transient, the treatment effects of thalamotomy are mainly associated with the regulation of connectivity that is directed toward the dentate nucleus in the cerebellum. More specifically, the sustained treatment effects of thalamotomy are associated with decreased effective connectivity from both the VL thalamus and SMA to the right dentate nucleus. Similarly, the CRST A posture scores were associated with effective connectivity among these regions. In other words, decreased posture scores (i.e., reduced symptom severity at rest) correlated with reduction in effective connectivity from both the VL and SMA to the dentate nucleus. In contrast to the posture scores, the action scores correlated with effective connectivity only from the SMA to the right dentate nucleus. Of note, the influence of symptom-related effective connectivity is more prominent in the posture scores than in the action scores. This may be explained by the fact that posture scores are measured at rest, a similar state for rs-fMRI.

Thalamotomy can affect the balance of activity in the dentate nucleus and non-VL thalamic nuclei. The increase in the effective connectivity from the non-VL thalamus to the dentate nucleus occurred in the context of a decrease in the effective connectivity from the VL and SMA to the dentate nucleus. This may suggest that an increase in effective connectivity from the non-VL thalamic nuclei to the dentate nucleus might be a complementary process to the reduction in VL activity induced by thalamotomy. The mechanisms underlying the reduction in self-inhibition (i.e., disinhibition) in the dentate nucleus remain unclear and require further study.

Since the thalamus is known to receive input from the dentate nucleus rather than feeding into the dentate nucleus, how does a thalamotomy affect connectivity from the thalamus to the dentate nucleus? It should be noted that effective connectivity can exist, even in the absence of direct anatomical projections, through polysynaptic connections. A closed motor loop, such as the thalamus–cortex–cerebellum-dentate nucleus circuit, might be a potential candidate circuit that is responsible for a polysynaptic effect. This reverse directional result was found in a study of DCM for deep brain stimulation (39), which demonstrated the existence of effective connectivity from the Vim to the cerebellum during deep brain stimulation. Neural degeneration in the distant white matter from the Vim nucleus might be an evidence for the circuitry changes that result from focal damage in the Vim (4). Wintermark and colleagues (4) found longitudinal degeneration (decreasing fractional anisotropy of DTI) in thalamic regions (and other distant regions) that belonged to motor circuits in patients with essential tremors after thalamotomy performed with MRgFUS. Distal circuitry changes have also been reported in functional connectivity metrics observed in our previous study (5).

Connectivity changes in circuits from non-VL thalamic nuclei to the dentate nucleus might also be mediated by the indirect pathway, through the thalamocortical (non-motor system)–cerebellar circuit. This interpretation is partly supported by a study that showed that tremor symptoms in patients with Parkinson’s disease correlated positively with the connectivity between the dentate nucleus and the bilateral cerebellar posterior lobes but correlated negatively with the connectivity between the dentate nucleus and the prefrontal cortex, to which non-VL thalamic nuclei project (40). These observations suggest that thalamotomies that are performed at the Vim may increase the effective connectivity from the non-VL thalamus to the dentate nucleus by this polysynaptic thalamocortical–cerebellar circuit.

Methodologically, we have focused on effective connectivity analyses instead of functional connectivity analyses, which are based on the temporal synchrony among endogenous BOLD fluctuations at rest (41). However, temporal synchrony can arise from a stimulus-locked common input or stimulus-induced oscillations through polysynaptic connections (42), and therefore does not provide information about the directed casual influences among brain regions. To overcome this limitation, we used DCM, which takes into account both the neuronal activity and the hemodynamic responses in the modeling of rs-fMRI (8).

Although we can estimate DCM at each session of each subject, group-level longitudinal analysis of effective connectivity is not a trivial issue. Conventionally, group-level generalization of DCM results require Bayesian model comparison and model averaging steps across sessions and subjects to determine a common (winning) model, followed by a conventional statistical analysis of model parameters (20). In this study, for group-level inference of longitudinal changes in effective connectivity, we adopted a general PEB approach (18, 19) and conducted a novel two-level PEB analyses that comprised between-session (within each individual) and between-subject group effects. We used empirical priors from the second (individual) level to iteratively optimize posterior densities over parameters at the first (session) level. This iterative approach finesses the local minima problem inherent in the inversion of non-linear and ill-posed models; thus, it provides more robust and efficient estimates of within and between-session effects (18, 43). Group-level inferences can then proceed using the same technology; in which group means provide empirical priors for parameters at the level below. As noted above, the PEB scheme allows for parametric random effects on connection strengths, between sessions or subjects, in contrast to random effects on models *per se* (20). This is clearly advantageous in the quantitative analysis of effective connectivity changes in terms of longitudinal mixed effects.

It should be noted that this study was based on resting-state responses, which may not be directly compatible with equivalent DCM studies during motor performance. Although resting-state (intrinsic) connectivity is known to be associated with task-related brain involvement (41, 44–47), discrepancies may be disclosed with detailed comparative analysis; for example, task-related brain activity may be a non-linear mixture of resting-state components (48, 49). Analysis of motor task-related data could supplement our understanding of the neural mechanisms that underwrite tremor and effects of thalamotomy on neural circuitry. In this study, we did not observe a significant shift in the directed connectivity from the thalamus to the cortical motor area. This may be partly due to the individual differences in the degree of changes in intrinsic connectivity from the thalamus to the motor cortex caused by the thalamotomy. In addition, it may be possible that the compensatory signals in the VL enhance or maintain signaling to the motor cortex.

In summary, using multilevel PEB of spectral DCM for longitudinal resting-state fMRI data, we have demonstrated that a focal lesion in the Vim of the thalamus induced by minimally invasive MRgFUS results in long-lasting symptom-related alterations in the effective connectivity of the dentate nucleus in the motor circuit. This study demonstrates the efficacy of multilevel PEB of spectral DCM for quantifying longitudinal changes in connectivity in humans using non-invasive techniques.

## Ethics Statement

All patients provided written informed consent before procedures, and this study received full ethics approval from the Korean Food and Drug Administration (KFDA) and Institutional Review Board of Yonsei University Severance Hospital and the Declaration of Helsinki (World Medical Association, 1964, 2008).

## Author Contributions

H-JP and JC designed the project; CP, CJ, and WC conducted experiments; H-JP, CP, and CJ analyzed the data; H-JP, CP, JC, KF, AR, and PZ wrote the manuscript.

## Conflict of Interest Statement

The authors declare that the research was conducted in the absence of any commercial or financial relationships that could be construed as a potential conflict of interest.
